# *Candida dubliniensis* as a Cause of Chronic Meningitis in a 3-Year-Old Boy with Acute Lymphoblastic Leukemia

**DOI:** 10.3390/pediatric18020055

**Published:** 2026-04-12

**Authors:** Adrianna Ćwiertnia, Laura Chuchla, Tomasz Ociepa

**Affiliations:** 1 Doctoral School, Pomeranian Medical University in Szczecin, Żołnierska 54, 71-210 Szczecin, Poland; adrianna.cwiertnia@gmail.com; 2 Department of Pediatrics and Hemato-Oncology, Pomeranian Medical University in Szczecin, Unii Lubelskiej 1, 71-252 Szczecin, Poland; tomasz.ociepa@pum.edu.pl

**Keywords:** acute lymphoblastic leukemia, antifungal susceptibility testing, *Candida dubliniensis*, child, chronic meningitis, fluconazole, intrathecal liposomal amphotericin B, therapeutic drug monitoring, voriconazole

## Abstract

*Candida dubliniensis* is an opportunistic yeast closely related to *Candida albicans* and an uncommon cause of central nervous system (CNS) infection. While isolates are often susceptible to azoles, reduced susceptibility or acquired resistance may occur, making species identification and antifungal susceptibility testing clinically relevant. We report a 3-year-old boy with Philadelphia chromosome-positive B-cell precursor acute lymphoblastic leukemia (ALL) in hematologic remission who developed chronic meningitis during maintenance chemotherapy. The initial presentation was non-specific (marked somnolence without fever or meningeal signs) and lumbar puncture performed to exclude CNS relapse revealed neutrophil-predominant pleocytosis and elevated protein; the cerebrospinal fluid (CSF) culture grew *C. dubliniensis*. Treatment with intravenous liposomal amphotericin B followed by prolonged fluconazole led to clinical improvement and sterile CSF. Six months later, progressive gait disturbance, limb pain, and episodic severe headaches recurred; repeat CSF cultures again yielded *C. dubliniensis*, with a changed susceptibility profile. Spine MRI demonstrated leptomeningeal enhancement involving the cauda equina nerve roots. Intravenous voriconazole with therapeutic drug monitoring was initiated and combined with intrathecal liposomal amphotericin B (seven doses, dose-escalated up to 3 mg), which was well tolerated and associated with rapid neurologic improvement, CSF sterilization, and radiologic resolution. At 12 months of follow-up, the patient remained infection-free and in leukemia remission. This case highlights that *C. dubliniensis* chronic meningitis may present subtly yet progress, requiring repeated CSF cultures with susceptibility testing; intrathecal liposomal amphotericin B can be a safe and effective adjunct to systemic therapy in refractory or recurrent disease.

## 1. Introduction

Acute lymphoblastic leukemia (ALL) is the most common childhood cancer [1]. In Poland, from 210 to 280 children are diagnosed each year, and the highest incidence occurs between the ages of 1 and 4 [2]. Less than 5% of children with ALL harbor a BCR-ABL1 gene fusion; in this group, patients receive tyrosine kinase inhibitor therapy in addition to conventional chemotherapy [3]. Due to the course of ALL and the weakened function of many organs, especially the immune system, patients are at particular risk of developing severe and difficult-to-treat complications [4]. Over the years, tremendous progress has been made in the treatment of ALL. However, ongoing complications, especially infections, are still the leading cause of death associated with the treatment of children with ALL, with the highest risk during the induction phase [5]. Special attention should be paid to invasive fungal infections (IFI), as they are one of the most common causes of infection-related deaths in patients with prolonged neutropenia [6]. The prevalence of IFI in ALL is reported to range from 4% to 35%, depending on the era, chemotherapy protocol, risk categories, and antifungal prophylaxis. Although the true incidence of IFIs is difficult to estimate, it has increased in recent years due to advances in diagnostic methods, intensive chemotherapy, prolonged neutropenia, and increased use of central catheters [7]. Central nervous system involvement at the time of diagnosis of acute lymphoblastic leukemia (ALL) occurs in approximately 5% of patients and is associated with an adverse prognosis [8]. In patients with ALL, neurological complications are relatively common and result from both the disease itself and, more frequently, its treatment. The incidence of neurological complications in ALL varies between 3% and 13% depending on different studies [9].

*Candida dubliniensis* is an opportunistic yeast pathogen and is usually resistant to fluconazole [10]. This species colonizes the oral cavity and exhibits a phenotype similar to that of *Candida albicans* [11,12]. Chronic meningitis caused by *C. dubliniensis* is rare [13]. It has only been described in very few cases.

We report on a case of a 3-year-old boy with Ph-positive acute lymphoblastic leukemia and chronic *C. dubliniensis* meningitis diagnosed during the maintenance phase of leukemia treatment.

## 2. Case Report

The 3-year-old patient had been under the care of the Department of Pediatrics, Hemato-Oncology and Gastroenterology in Szczecin, Poland, since April 2021 due to a diagnosis of B-cell precursor acute lymphoblastic leukemia Ph-positive (ALL-BCP, Ph+).

Following the diagnosis, treatment was started according to the EsPhALL2017/COGAALL1631 protocol, which also included the use of a tyrosine kinase inhibitor (imatinib). The induction therapy, consolidation, and delayed intensification protocols had to be interrupted several times due to serious complications, including peritonitis, severe sepsis, and ileus followed by gastrointestinal perforation, which necessitated the creation of a small bowel stoma. A follow-up bone marrow examination showed complete hematologic remission and negative minimal residual disease (MRD) assessed by PCR. Therefore, the patient was classified as standard risk (SR) and had continued intensive chemotherapy.

**Figure 1:**
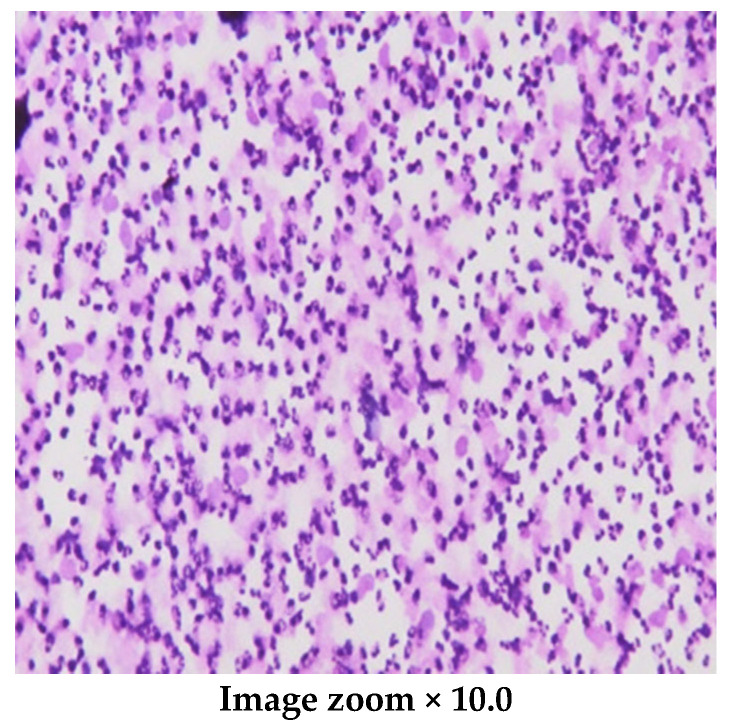
The microscopic image of CSF cells stained with May–Grunwald–Giemsa (MGG).

Less than a year after diagnosis, the patient was admitted to the department due to severe diarrhea (>15 stools/day), decreased fluid intake, weakness, excessive sleepiness, and abdominal pain. On physical examination, the patient appeared well. His temperature was 36.8 °C, pulse was 110 beats per minute, blood pressure was 90/65 mmHg, respirations were 26 breaths per minute, and his oxygen saturation was 98% while breathing ambient air. His pupils were symmetrical. There was no evidence of neck rigidity, Kernig’s, or Brudzinski’s signs. Laboratory tests showed mild hypokalemia, slightly elevated liver enzymes, agranulocytosis, and profound thrombocytopenia. Inflammatory parameters, blood, and stool microbiological tests were negative. Initial treatment included intravenous hydratation, antidiarrheals, analgesics, and platelet transfusions. Due excessive sleepiness observed in the patient, a lumbar puncture was performed to confirm the diagnosis. The analysis of cerebrospinal fluid (CSF) showed a high protein concentration and increased number of cells with a predominance of neutrophils. The microscopic image of CSF cells is presented in Figure 1, and the laboratory data are presented in Table 1.

CSF culture was positive for *Candida dubliniensis*. Antifungal susceptibility testing revealed the minimum inhibitory concentrations (MICs) for amphotericin B and fluconazole of 0.023 mg/L and 0.38 mg/L, respectively, which is why the combination of antifungal agent consisting of intravenous liposomal amphotericin B (AmBisome) at a dose of 5 mg/kg/d and fluconazole at a dose of 10 mg/kg/d was introduced.

As a potential source of infection, the port-a-cath (vascu-port) was removed. No microbiological findings were reported on the removed central venous catheter.

The patient’s general condition improved, symptoms resolved, and a follow-up CSF culture returned negative. Liposomal amphotericin B was terminated after 14 days, and treatment with fluconazole was continued for seven weeks in total. Maintenance chemotherapy, as scheduled, was continued with good tolerance.

Six months later, he was once again admitted to the clinic on an emergency basis due to increasing gait disturbances, lower limb pain, and episodic severe headaches associated with agitation, screaming, and self-aggression.

On physical examination, the patient appeared relatively well. His temperature was 36.6 °C, pulse was 120 beats per minute, blood pressure was 90/60 mmHg, respirations were 24 breaths per minute, and his oxygen saturation was 97% while breathing ambient air. His pupils were symmetrical, and there was again no evidence of neck rigidity or Kernig’s or Brudzinski’s signs. Laboratory tests upon admission showed a low CRP level and moderate neutropenia. An extensive diagnostic evaluation was performed, including MRD assessment, which was negative, and a lumbar puncture. CSF analysis revealed increased cellularity with neutrophil predominance. These data are present in Table 1.

The patient was empirically started on ceftazidime and fluconazole. The CSF culture was positive again for *Candida dubliniensis*, so treatment was modified, and according to the susceptibility testing (MIC 0.047 mg/L), intravenous voriconazole with therapeutic drug monitoring (TDM) was introduced. It should be noted that MIC for amphotericin B and fluconazole were 0.47 mg/L and 2 mg/L, respectively.

**Figure 2:**
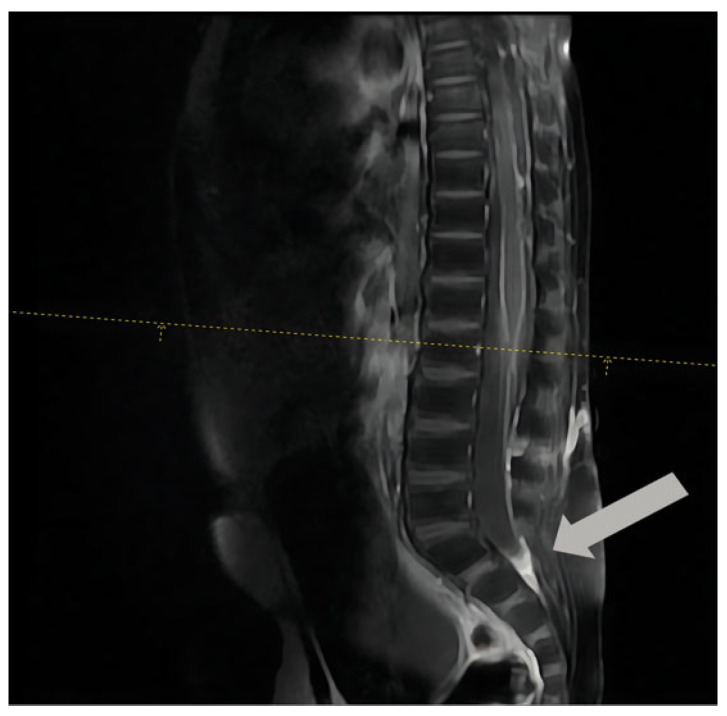
Magnetic Resonance Imaging shows distinct leptomeningeal enhancement along the lumbar spine and cauda equina nerve roots, as indicated by an arrow.

Brain and spine magnetic resonance imaging (MRI) was done to identify a possible source of the extremely rare recurrent infection. It revealed meningeal spine enhancement, including the cauda equina’s nerve roots. The patient’s spine MRI is shown in Figure 2.

Due to MRI findings suggestive of a possible residual CNS infection, the treatment was modified again. As a second agent, intrathecal liposomal amphotericin B at an initial daily dose of 1 mg every second day was introduced. The dose was titrated by 1 mg every 48 h to a maximum daily dose of 3 mg.

**Figure 3:**
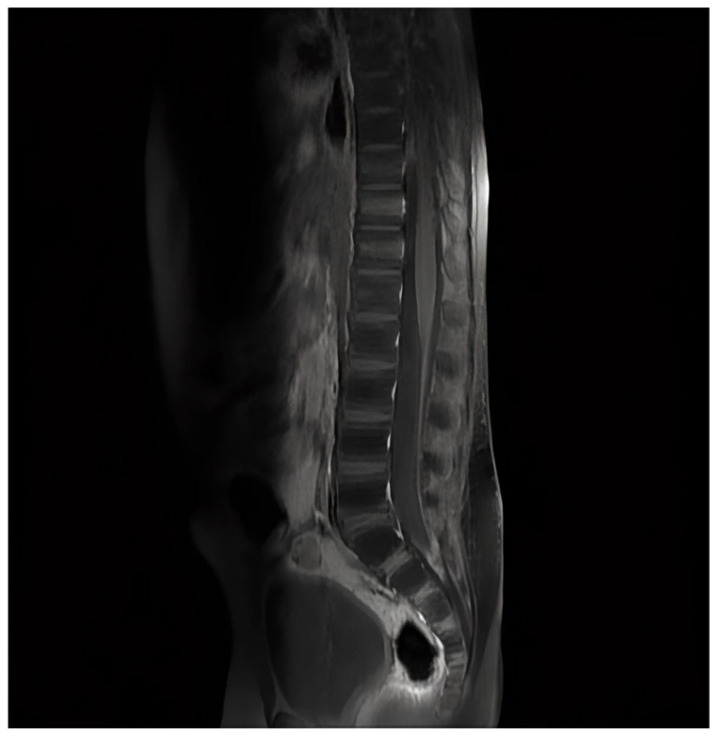
Follow-up Magnetic Resonance Imaging shows complete resolution of the previously noted meningeal enhancement.

**Figure 4:**
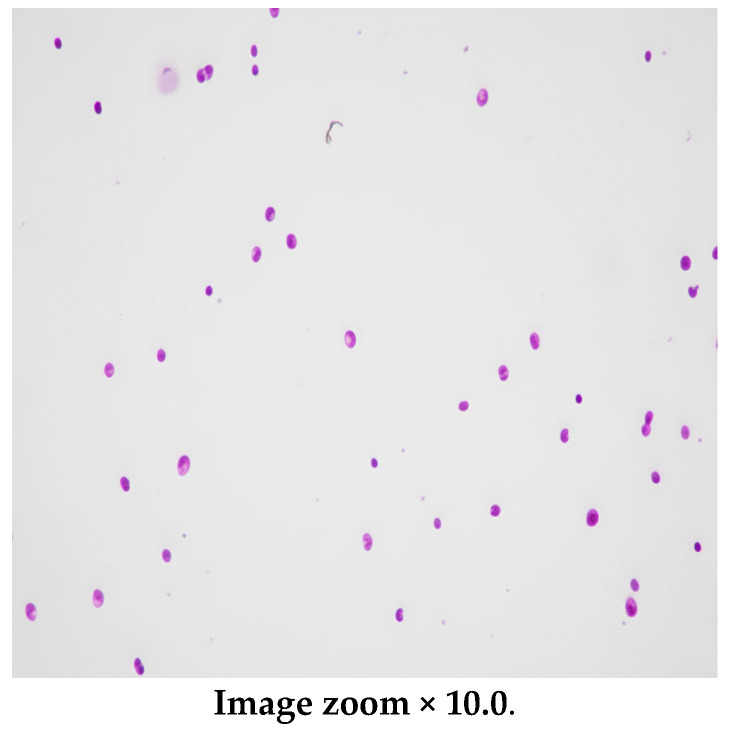
The microscopic image of CSF cells stained with May–Grunwald–Giemsa (MGG).

**Figure 5:**
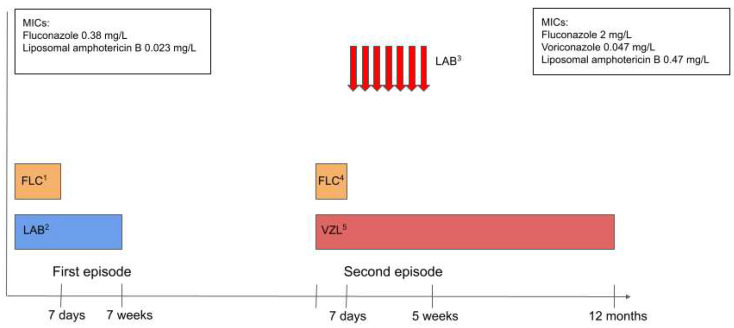
Timeline of the antifungal treatment. ^1^ Fluconazole 10 mg/kg/d i.v., ^2^ Liposomal amphotericin B 5 mg/kg/d i.v., ^3^ Intrathecal liposomal amphotericin B: initial daily dose of 1 mg every second day (titrated by 1 mg every 48 h to a maximum daily dose of 3 mg)—indicated by arrows, ^4^ Fluconazole 10 mg/kg/d i.v., ^5^ Voriconazole 9 mg/kg p.o.

The patient’s condition rapidly improved, and neurological symptoms abated. The patient received seven intrathecal doses of liposomal amphotericin B. The treatment with voriconazole was continued for 12 months and was well tolerated. The control spine MRI revealed the resolution of the initially observed meningeal enhancement. This is shown in Figure 3. Repeated CSF analysis revealed a gradually decreasing neutrophil count. The microscopic image of CSF cells is presented in Figure 4. CSF culture became negative after 14 days of treatment. Timeline of the antifungal treatment is presented in Figure 5.

## 3. Discussion

In the presented case, clinical improvement and sterile CSF were achieved during the first episode of treatment with intravenous liposomal amphotericin B and extended fluconazole. Intravenous voriconazole with therapeutic drug monitoring was initiated at recurrence and combined with intrathecal liposomal amphotericin B (7 doses in total, dose-escalated from 1 mg to 3 mg), which was well tolerated and associated with rapid radiologic resolution, CSF sterilization, and neurologic improvement. The patient was still free of infections and in remission from leukemia after a 12-month follow-up.

This case shows that intrathecal liposomal amphotericin B, although off-label, can be a safe and effective adjuvant to systemic therapy in cases of resistant or recurrent *C. dubliniensis* chronic meningitis, which may start slowly but progress to the point where repeated CSF cultures with susceptibility testing and intensive antifungal treatment are required.

The overall incidence of invasive fungal disease in children with ALL ranges from 8 to even 40 cases per 100 children [14,15,16]. Invasive candidiasis (IC), particularly candidemia, may be responsible for up to 35% of all IFD, and meningitis caused by *Candida* spp. may be associated with a very high mortality rate [14,17]. Common risk factors for IFD include the use of broad-spectrum antibiotics, chemotherapy, steroid exposure, immunosuppressive treatment, including profound neutropenia, parenteral nutrition, and hematopoietic or solid organ transplantation [6,17]. Antifungal prophylaxis may minimize the incidence of IFD in hematological patients [16], including children [6]. International guidelines recommend systemic active antifungal prophylaxis when the incidence of IFD is >10% [18,19]. Although Candida species are the leading cause of IFD in children, *C. dubliniensis* represents only a small proportion of *Candida* isolates and is far less common in this group of patients. On the other hand, the number of documented cases has increased significantly over the years [20]. However, due to its rarity, the risk factors, prevalence, and prognosis are not fully understood. Suggested risk factors for *Candida dubliniensis* infections include the use of intravascular catheters, broad-spectrum antibiotic therapy, immunocompromised states, including human immunodeficiency virus (HIV) infection, chemotherapy, and solid organ transplantation [21]. A recent Danish nationwide study reported a doubling in the incidence of *C. dubliniensis* fungemia from 2012 to 2015 [17]. Moreover, recent reports described *Candida dubliniensis* as a potential cause of catheter-associated bloodstream infections, endocarditis, and endophthalmitis [22,23]. To the best of our knowledge, we are reporting the first case of *C. dubliniensis* chronic meningitis in a child with ALL.

The patient presented with numerous risk factors for invasive fungal infection. These were intensive chemotherapy for acute leukemia, the presence of a central line, the use of parenteral nutrition, prolonged neutropenia, broad-spectrum antibiotics, and compromised gastrointestinal integrity caused by previous infections and abdominal surgery.

Given the rarity of chronic *C. dubliniensis* meningitis in children, specific diagnostic and management guidelines are lacking. Additionally, little is known about the prognosis for children with ALL complicated by chronic *Candida dubliniensis* meningitis.

The management of invasive *Candida* spp. infections in children includes prompt initiation of antifungal therapy and immediate control of the infection source by removing indwelling intravenous catheters. The recommended treatment duration for uncomplicated candidemia is 14 days after blood cultures have become sterile, and the patient has recovered from neutropenia [20]. For invasive candidiasis, the resolution of predisposing conditions usually influences the duration of treatment. The European Conference on Infections in Leukemia (ECIL-8) recommends echinocandins or liposomal amphotericin B at a dose of 3 mg/kg/d as first-line treatment for invasive *Candida* spp. infections, even before species identification [20]. However, the diagnosis and treatment of CNS candidiasis differ from those of candidemia. First, the diagnosis may be challenging because the isolation of Candida from CSF is uncommon. Second, treatment should consist of drugs that effectively penetrate the CNS.

In our case, the diagnosis of meningitis caused by *Candida dubliniensis* resulted from a positive CSF culture. However, it should be noted that symptoms of potential CNS infection were relatively subtle in the reported case. All meningeal signs were negative, and the patient was afebrile. Moreover, any new neurological symptom in a patient with leukemia may be suggestive of CNS relapse. That is why a lumbar puncture was performed. Fortunately, CSF analysis is used not only to diagnose CNS leukemia relapse, but also meningitis. The predominance of neutrophils in the CSF and the absence of blasts suggest a bacterial or fungal infection. It is worth mentioning that we did not find elevated inflammatory markers in our case. It may be explained by CRP’s low sensitivity in predicting severe infection, or by the mild course of *Candida dubliniensis* infection.

Considering that echinocandins have only minimal CNS penetration, we decided to treat our patient with the use of liposomal amphotericin B at a higher-than-recommended dose for the treatment of candidemia. We also introduced fluconazole, which has excellent CNS penetration and may be a reasonable alternative for patients without previous fluconazole prophylaxis [24].

The treatment choice was also based on the isolate’s antifungal susceptibility test, which revealed MICs of 0.023 mg/L for liposomal AmB and 0.38 mg/L for fluconazole. Moreover, at least a few publications reported that such a combination of drugs is effective in chronic meningitis caused by *Candida dubliniensis* [13,25]. *Candida* spp. biofilm formation may be responsible for treatment failure in patients with candidiasis/candidemia [26]. The port-a-cath, a central venous access device, was removed almost immediately because it was initially suspected to be a potential source of the infection and a hindrance to treatment. Although the CSF culture became negative after 14 days of treatment, given the high-risk patient, we continued fluconazole for a total of 7 weeks. The recurrence of infection after such a long interval (specifically, 6 months in the reported case) is unusual, even in a patient with leukemia. This *C. dubliniensis* meningitis recurrence occurred during the maintenance phase of treatment, which is typically well-tolerated and has a lower absolute risk of severe infection. The absence of established treatment standards may contribute to recurrent infections. In this case, the duration of therapy for the initial episode might have been too short. Furthermore, immunosuppression and cytostatic toxicity may negatively impact the patient’s mucous membranes, which are key components of the immune defense, thereby increasing the risk of recurrence. It is also possible that the penetration of the administered drugs was inadequate. The second-line treatment was based on the antifungal susceptibility test of the *Candida dubliniensis* isolate, which showed the lowest MIC for voriconazole (0.047 mg/L). MICs for liposomal AmB and fluconazole were 0.47 mg/L and 2 mg/L, respectively, and were higher than those reported in previous tests. According to ECIL-8, voriconazole is a secondary option after liposomal amphotericin B and echinocandins for first-line treatment of candidemia [26,27]. It shows activity on yeasts and some molds. Due to its good penetration across the blood–brain barrier, it is recommended as a primary therapy for CNS aspergillosis and a second-line option for other CNS infections [24,26,28]. In addition, compared with the intravenous formulation, the oral formulation of voriconazole also shows high bioavailability and only moderate protein binding [24]. This makes the oral formulation of voriconazole attractive because it may be used in outpatient care as a maintenance treatment for CNS infection at follow-up. Due to recurrent infection caused by a rare yeast, a brain and spine MRI was done. It revealed meningeal spine enhancement, which may be suggestive not only of chronic meningitis. Although there are no guidelines on the use of intrathecal amphotericin B in cerebral fungal infections, its efficacy and good tolerability have been reported in patients with fungal meningitis, including children [29,30,31,32]. Intrathecal administration of amphotericin B may be effective and complementary to intravenous therapy for fungal meningitis [29]. We used intrathecal liposomal amphotericin B seven times, with excellent tolerability. No adverse events, including headache or nausea, were recorded in our patient during intrathecal administration of liposomal amphotericin B at a maximum dose of 3 mg.

## 4. Conclusions

Chronic meningitis caused by *Candida dubliniensis* in children with acute leukemia is rare and may be a diagnostic and therapeutic dilemma. *Candida dubliniensis* chronic meningitis may be associated with relatively mild but progressive symptoms and no systemic features. CSF fungus cultures and susceptibility tests are crucial for prompt diagnosis and proper treatment choice. Although systemic treatment based on the susceptibility test and drug brain penetration is a standard of care, the type and duration of therapy in immunocompromised patients have yet to be clarified. Intrathecal administration of amphotericin B may be a safe and effective adjunctive to chronic *Candida dubliniensis* meningitis intravenous therapy.

## Figures and Tables

**Table 1:** Laboratory data taken at first episode and at recurrence.

Parameter	Values		References Values *
	First Episode	Recurrence	
Leucocytes (k/µL)	0.85	2.35	4.50–13.00
Erythrocytes (M/µL)	3.46	3.30	3.90–5.00
Hemoglobin (g/dl)	10.5	12.3	10.9–14.2
Hematocrit (%)	28.9	36.4	34.0–41.0
Platelets (k/µL)	42	164	210–490
Neutrophiles (k/µL)	0.16	no data	35.0–55.0
Lymphocytes (k/µL)	0.35	0.32	25.0–50.0
Monocytes (k/µL)	0.30	0.58	0.30–2.00
CRP ** (mg/L)	0.64	4.21	<5.00
Sodium (mmol/L)	136–135		135–145
Potassium (mmol/L)	2.06–4.06		3.50–5.50
Total calcium (mmol/L)	2.08–2.33		2.19–2.69
Ionized calcium (mmol/L)	1.28–1.25		1.12–1.32
Phosphate (mmol/L)	0.76–123		1.00–1.95
Magnesium (mmol/L)	0.82–0.84		0.62–0.95
Creatinine (mg/dL)	<0.17–0.21		0.24–0.47
Albumin (g/dL)	3.03–nd		3.80–5.40
AST ^1^ (U/L)	23–28		0–31
ALT ^2^ (U/L)	20–38		0–71
Total bilirubin (mg/dL)	0.79–0.28		0.00–1.10
Uric acid (mg/dL)	1.8–nd		3.4–7.0
Ferritin (µ/dL)	3041–1933		6.00–67.00
Iron (µ/dL)	197–nd		59–158
CSF *: Color	Colorless–Colorless		Colorless
CSF: Clarity	Clear–Clear		Clear
CSF: Blood clot	Negative–Negative		Negative
CSF:Nonne-Apelt’s reaction	Negative–Negative		Negative
CSF:Pandy’s reaction	Negative–Negative		Negative
CSF:Proteins (mg/dL)	30.3–126		15–45
CSF:White blood cells (cells/µL)	Lymphocytes—61 Lymphocytes—155 Granulocytes—405 Granulocytes—177		0–5

^1^ AST—aspartate transaminase, ^2^ ALT—alanine transaminase. ** CRP—C-reactive protein, * CSF—cerebrospinal fluid, nd—no data.

## Data Availability

The original contributions presented in this study are included in the article. Further inquiries can be directed to the corresponding author.
